# Exercise-Induced Fitness Changes Correlate with Changes in Neural Specificity in Older Adults

**DOI:** 10.3389/fnhum.2017.00123

**Published:** 2017-03-16

**Authors:** Maike M. Kleemeyer, Thad A. Polk, Sabine Schaefer, Nils C. Bodammer, Lars Brechtel, Ulman Lindenberger

**Affiliations:** ^1^Center for Lifespan Psychology, Max Planck Institute for Human DevelopmentBerlin, Germany; ^2^Computational and Cognitive Neuroscience Laboratory, Department of Psychology, University of MichiganAnn Arbor, MI, USA; ^3^Institute of Sport Science, Saarland UniversitySaarbruecken, Germany; ^4^Berlin Academy of Sports MedicineBerlin, Germany; ^5^European University Institute, San Domenico di Fiesole (FI)Italy

**Keywords:** aging, fitness, physical exercise, neural specificity, multivariate pattern analysis

## Abstract

Neural specificity refers to the degree to which neural representations of different stimuli can be distinguished. Evidence suggests that neural specificity, operationally defined as stimulus-related differences in functional magnetic resonance imaging (fMRI) activation patterns, declines with advancing adult age, and that individual differences in neural specificity are associated with individual differences in fluid intelligence. A growing body of literature also suggests that regular physical activity may help preserve cognitive abilities in old age. Based on this literature, we hypothesized that exercise-induced improvements in fitness would be associated with greater neural specificity among older adults. A total of 52 adults aged 59–74 years were randomly assigned to one of two aerobic-fitness training regimens, which differed in intensity. Participants in both groups trained three times a week on stationary bicycles. In the low-intensity (LI) group, the resistance was kept constant at a low level (10 Watts). In the high-intensity (HI) group, the resistance depended on participants’ heart rate and therefore typically increased with increasing fitness. Before and after the 6-month training phase, participants took part in a functional MRI experiment in which they viewed pictures of faces and buildings. We used multivariate pattern analysis (MVPA) to estimate the distinctiveness of neural activation patterns in ventral visual cortex (VVC) evoked by face or building stimuli. Fitness was also assessed before and after training. In line with our hypothesis, training-induced changes in fitness were positively associated with changes in neural specificity. We conclude that physical activity may protect against age-related declines in neural specificity.

## Introduction

Pictures of faces, houses, and many other stimulus categories elicit distinguishable patterns of neural response in ventral visual cortex (VVC). For example, using functional magnetic resonance imaging (fMRI), Haxby et al. (2001) found distinct neural activation patterns in response to eight stimulus categories within ventral temporal cortex. After being trained on a subset of activation patterns, machine learning classifiers can often decode the stimulus category associated with novel patterns (Haynes and Rees, 2006; Norman et al., 2006). And the more distinctive, or specific, the patterns are, the more accurately a classifier will be able to predict the stimulus category from neural activity, so classifier accuracy is a natural way to estimate neural specificity.

Evidence suggests that neural specificity declines with increasing age (Grady et al., 1994; Park et al., 2004) and reduced neural specificity is associated with lower cognitive performance in a variety of cognitive tasks in older adults (Park et al., 2010). But why does neural specificity decline with age? Age-related declines in neurotransmitter function and neuromodulation have been suggested as underlying mechanisms. For example, the number of dopamine (DA) neurons (Bäckman et al., 2006), as well as DA receptor levels (Inoue et al., 2001) show pronounced and widespread decrements with advancing adult age. Neurocomputational models predict that attenuated neuromodulation lowers a cell’s responsivity and leads to less differentiated neural responses to different stimuli (i.e., less distinct neural representations), which in turn would explain age-related deficits across a wide range of cognitive domains (Li and Sikström, 2002).

On the other hand, regular physical activity has repeatedly been shown to preserve cognitive abilities in old age (for reviews see Bherer et al., 2013; Voss et al., 2013). Evidence from animal research suggests that exercise induces an upregulation of DA (Sutoo and Akiyama, 2003; Poulton and Muir, 2005; Foley and Fleshner, 2008), possibly by stimulating DA synthesis through a calcium/calmodulin-dependent system. Consistent with this hypothesis, Ruscheweyh et al. (2011) found increased plasma concentrations of DA after 6 months of physical training in older humans. Moreover, Stroth et al. (2010) showed that young adults with a genotype associated with lower DA levels (val/val COMT gene homozygotes) exhibited greater cognitive improvements after a 4-month exercise intervention compared with other genotypes (met carriers). Since the relationship between DA and cognitive performance seems to follow an inverted U-shape, Stroth et al. (2010) hypothesized that exercise may optimize central DA availability. This especially benefits those individuals with suboptimal initial levels, as it moves them further up the curve. Similarly, a cross-sectional study with older adults showed that val/val homozygotes benefitted most from better fitness in terms of Flanker task performance. The latter result suggests that high fitness levels may compensate for being a COMT val/val homozygote (Voelcker-Rehage et al., 2015).

Taken together, the two lines of research suggest that neural specificity may provide a potential mechanism for beneficial effects of exercise in preserving cognitive functions in older adulthood. If so, improvements in exercise-induced fitness should be associated with more positive changes in neural specificity.

We are aware of four previous intervention studies that looked at exercise-related changes in BOLD activation (Colcombe et al., 2004; Voss et al., 2010; Voelcker-Rehage et al., 2011; Maffei et al., 2017). All four of them focused on changes in *neural* *efficiency*, that is, more efficient usage of brain networks, which is typically reflected in a reduced BOLD signal, and in greater functional connectivity with increasing cognitive load. The results of these studies suggest that an aerobic fitness training as opposed to an anaerobic fitness training or a passive control improves neural efficiency during task performance, as reflected by maintained (Maffei et al., 2017) or even reduced (Colcombe et al., 2004; Voelcker-Rehage et al., 2011) BOLD activation in task-relevant areas, as well as by strengthened functional connections within the default mode and frontal executive networks (Voss et al., 2010). In contrast to these former studies, which focused on exercise-induced changes in neural efficiency, the present study investigates exercise-induced changes in *neural specificity*, that is, the distinctiveness of specific stimulus-evoked neural activation patterns, irrespective of BOLD signal strength. Note that it is possible to observe changes in neural efficiency without changes in neural specificity, such as when two different stimuli elicit smaller, more efficient activation after the intervention that are not accompanied by reductions in activation overlap. Likewise, one may observe changes in neural specificity without changes in neural efficiency, such as when two stimuli elicit more distinct activation patterns after the intervention that are not accompanied by reductions in BOLD signal strength.

To test whether exercise-induced fitness improvements translate to neural specificity we used fMRI and multivariate pattern analysis (MVPA) within an exercise-dose-response paradigm. Elderly participants were randomly assigned to training regimens with differing levels of intensity. Before and after the 6-month training phase, participants performed a graded maximal exercise test to assess their training-related fitness improvements as well as a passive viewing task while functional brain images were acquired. As in earlier work (see Carp et al., 2011), the distinctiveness of neural activation patterns in response to different stimulus categories served as an index of neural specificity. To examine the hypothesized association between fitness and neural specificity, we correlated changes in fitness with changes in neural specificity.

## Materials and Methods

### Participants

The total sample consisted of 52 community-dwelling older adults aged 59–74 (mean 65.95 ± 4.36, 20 males). All participants met the following inclusion criteria: (1) age range in years between 59 and 75; (2) physical inactivity prior to study enrollment (MET < 40 based on the German version of the compendium of physical activities); (3) MMSE score ≥ 26; (4) free of neurological, psychiatric, and cardiovascular diseases; (5) right-handed; (6) no contraindication for heart-rate controlled exercise training (e.g., no medication with beta blockers); (7) suitability for MR assessment (e.g., no magnetic implants, no claustrophobia). This study was carried out in accordance with the recommendations of the ethics committee of the German Psychological Society (DGP). All participants gave written informed consent in accordance with the Declaration of Helsinki and participated voluntarily. They were paid for study completion; training adherence was reinforced through a bonus system. Details on the recruitment can be found in previously published work based on the same study (Kleemeyer et al., 2016). Five participants were excluded from the MVPA analyses, four due to incomplete fMRI data, and one due to improper slice positioning at pretest. One participant was excluded from the fitness analyses due to problems in VO_2_max detection. Thus, correlation analyses were based on data from 46 participants.

### Design

Participants completed a 6-month fitness intervention with a comprehensive test battery before (pre) and after (post) the training. Please note that we confine ourselves to describing only those methods relevant for the scope of this article, that is, training, fitness assessment, and imaging procedures. Additional information can be found in Kleemeyer et al. (2016).

### Training

Participants were randomly assigned to a high-intensity (HI) or low-intensity (LI) training regimen subsequent to the pretest assessment. Groups were counterbalanced for age, sex, years of education, digit-symbol performance, and MMSE scores.

Participants in each of the two groups exercised in our lab on stationary bikes, three times a week for 55 min in each session, with a gradual increase during the first 3 weeks. Over the 6-month period, a total of 75 training sessions could be achieved. For the HI group, training intensity was calibrated to result in a heart rate at 80% of the individual’s ventilatory anaerobic threshold (Wasserman et al., 1990). In contrast, the LI group exercised at a constant resistance of 10 W. For the last 21 sessions, five intervals of 2 min each were integrated after 20 min of training in order to further increase variance in fitness gains. During these 2-min time windows, the LI group only increased the cadence from 60–70 to 80–90 cycles/min, while the HI group also increased the intensity to a resistance corresponding to 110% of the individual’s ventilatory anaerobic threshold. Training intensity was automatically controlled using the software custo cardio concept (custo med GmbH, Ottobrunn, Germany), with a staff member supervising compliance for each participant and each training session. Up to six participants exercised simultaneously, irrespective of intensity levels (e.g., HI and LI participants exercised together). The existence of different training regimens was conveyed only after termination of the study.

### Cardiovascular Fitness Assessment

Participants performed a graded maximal exercise test on a cycle ergometer to assess their cardiovascular fitness. The test started at 10 W, increased to 25 W after 2 min followed by 25 W increments every 2 min until total exhaustion or signs of cardiac or respiratory distress. A sports physician continuously monitored the cardiogram, oxygen uptake, heart rate, and blood pressure. We computed an aggregate measure of the maximum oxygen consumption at exhaustion (VO_2_max) and the oxygen consumption at the ventilatory anaerobic threshold (VO_2_AT) to obtain a more robust fitness measure. Therefore, data were *z*-transformed in a way that preserves mean differences between time points, namely by subtracting the common mean from pretest and posttest data. The aggregate fitness measure VO_2_ served as the outcome of the fitness assessment.

### MRI Data Acquisition and Preprocessing

During functional imaging, participants passively viewed face, house, or phase-scrambled images, following the procedures of Park et al. (2010). They completed two runs, each of which consisted of four blocks per stimulus category. During every block 15 images were shown for 2 s each, resulting in 30 s per block and 6 min per run. Stimuli were presented via *E*-prime (Psychology Software Tools, Pittsburgh, PA, USA) and displayed by a projection system.

Brain images were acquired on a Siemens TIM Trio 3T MRI scanner (Siemens, Erlangen, Germany). A conventional echo-planar MR sequence was used for functional acquisitions (TR = 2000 ms, TE = 30 ms, flip angle = 80°, FOV = 216 mm) encompassing 192 volumes per run and 36 slices per volume (slice thickness 3 mm). Slices were 72 × 72 matrices acquired parallel to the Corpus Callosum. A high-resolution T1-weighted MPRAGE (TR = 2500 ms, TE = 4.76 ms, TI = 1100 ms, flip angle = 7°, acquisition matrix = 256 × 256 × 176, 1 mm isotropic voxels) was also acquired to facilitate warping masks from MNI to individual subject space. Data were preprocessed using SPM12 (Wellcome Department of Cognitive Neurology, London, UK^1^). Functional images were realigned to the mean volume. The T1-weighted image was normalized to MNI space. The inverse normalization parameters were then applied to an AAL atlas based mask of VVC (including bilateral occipital cortices, inferior temporal cortices, and fusiform gyri). As a last step, the T1-weighted image was co-registered to the mean functional image, and the same parameters were applied to the mask such that all images mapped into the subject’s native space. No normalization, spatial smoothing or other transformation was applied to the functional images.

To obtain activation maps, we setup a General Linear Model (GLM) to estimate the response to each category relative to phase-scrambled control images. We defined a separate regressor for each experimental block, resulting in eight estimates of face-evoked activation and eight estimates of house-evoked activation. We also included six nuisance covariates per run in the GLM, modeling head translation and rotation.

### Multivariate Pattern Analysis

Since we were interested in the responses to face and house stimuli, we restricted the analysis to voxels within VVC. We applied MVPA using correlation analysis (see Haxby et al., 2001) on individual subject data separately for pretest and posttest. More precisely, we used the 16 coefficient estimates for faces and houses vs. phase-scrambled images from the GLM and extracted the activation pattern for voxels within VVC. Next, we computed the Pearson correlation within categories (i.e., correlating the face-evoked activation patterns from odd blocks pairwise with the face-evoked activation patterns from even blocks and the same for house-evoked activation patterns) and the Pearson correlation between categories (i.e., correlating the face-evoked activation patterns from odd blocks pairwise with the house-evoked activation patterns from even blocks and vice versa). To ensure a more normal distribution, correlation coefficients were transformed into Fisher’s *z*-values. Neural specificity was then defined as the difference between the mean within-category and between-category correlations.

### Statistical Analyses

Statistical analyses were performed using SPSS (IBMCorp., IBM SPSS Statistics, V22, Armonk, NY, USA). To assess effects on fitness and neural specificity, we used repeated-measures analysis of variance (ANOVA) with time point as a within-subject factor and training group as a between-subject factor. To examine whether changes in fitness were associated with changes in neural specificity, we performed a two-tailed Pearson correlation analysis across participants from both groups using the absolute difference (post-training minus pre-training) in fitness and the absolute difference (post-training minus pre-training) in neural specificity. The alpha level for all analyses was set to *p* = 0.05.

## Results

There were no baseline differences between participants in the two training groups with respect to age, years of education, MMSE, BMI, fitness, hormone replacement therapy, and treated hypertension (see Table 1 for means and standard deviations (SD) or proportion of participants, respectively). Also, training adherence did not differ reliably between the two groups (mean HI = 71.14, mean LI = 69.12, *t*_(44)_ = −1.088, *p* = 0.282).

**Table 1:** **Sample characteristics as a function of training group**.

Characteristic	High intensity (*N* = 21)		Low intensity (*N* = 25)		*p*
	*M*	*SD*	*M*	*SD*	
Age (years)	66.56	4.33	65.93	4.50	0.631
Education (years)	11.05	1.63	11.36	1.63	0.520
MMSE	29.48	1.08	28.88	1.48	0.132
BMI	26.12	4.21	25.18	4.05	0.444
VO_2_max (ml/min × kg)	21.39	5.14	22.06	5.79	0.683

	*N*	**%**	*N*	**%**	

Female	13	62	15	60	
Hormone replacement therapy	5	38	6	40	
Treated hypertension	3	14	2	8	

*Values are means (M) and standard deviations (SD), or number of participants (N) and percentage within group (%), respectively. Abbreviations: MMSE, Mini-Mental State examination; BMI, Body mass index*.

The intervention was associated with increasing fitness levels (*F*_(1,49)_ = 5.637; *p* = 0.022; $\eta_{\text{p}}^{2}$ = 0.103). However, HI and LI groups did not differ in mean fitness changes (*F*_(1,49)_ = 0.997; *p* = 0.323; $\eta_{\text{p}}^{2}$ = 0.020). Consequently, data were collapsed across treatment conditions when investigating exercise effects on neural specificity.

**Figure 1:**
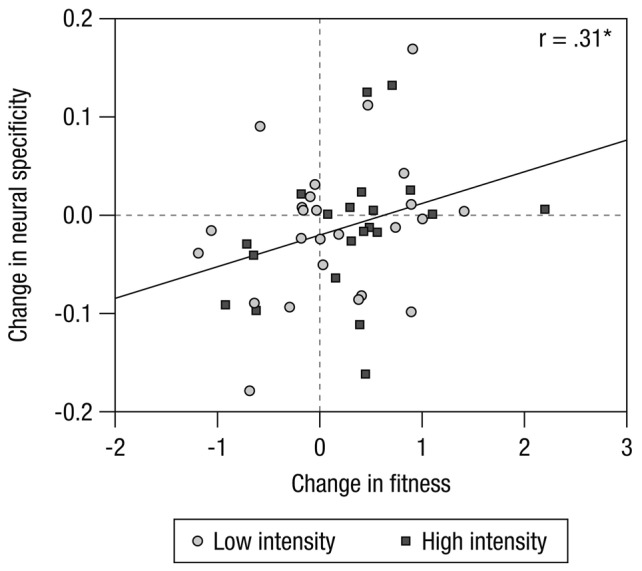
**Changes in fitness (aggregate measure of VO_2_max and VO_2_AT) are positively associated with changes in neural specificity.** Circles represent participants from the low-intensity (LI) exercise group, and squares represent participants from the high-intensity (HI) exercise group. Change scores refer to post-training minus pre-training differences; a score of zero signifies no change. *p < 0.05.

**Figure 2:**
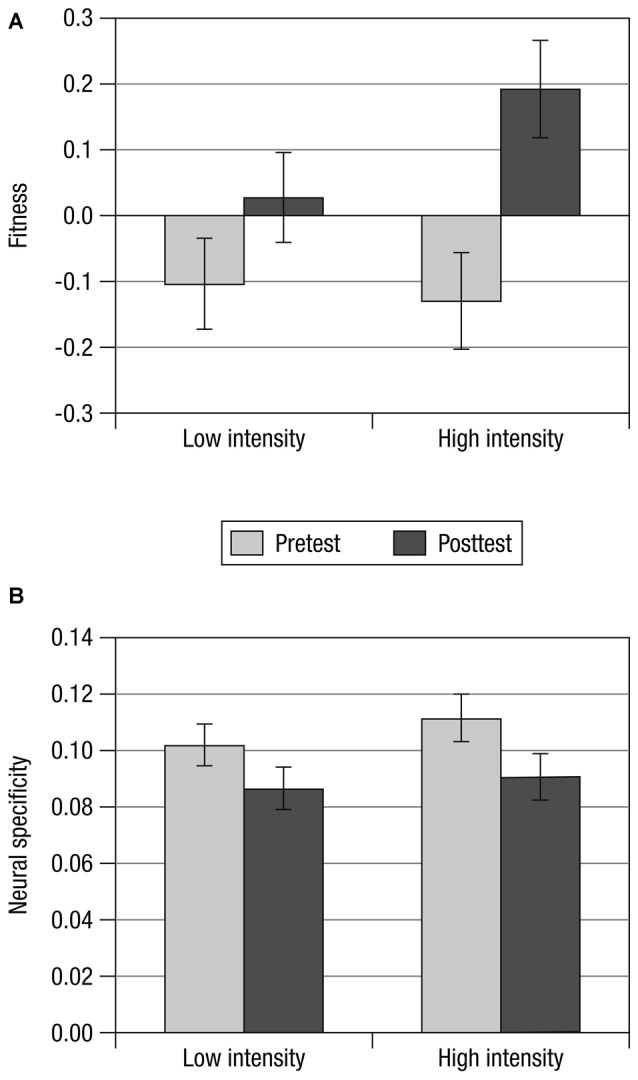
**Mean changes over the course of the training in (A)** fitness (aggregate measure of VO_2_max and VO_2_AT) and** (B)** neural specificity. Whereas fitness increased reliably from pretest (light gray) to posttest (dark gray), changes in neural specificity were not significantly different from zero. For both measures, changes in the LI training group (left) were not reliably different from changes in the HI training group (right). Error bars depict the standard error after removing between-person variability (Cousineau and O’Brien, 2014).

As predicted, greater changes in fitness were associated with greater changes in neural specificity (*r*_(46)_ = 0.310, *p* = 0.036, *R*^2^ = 0.096), irrespective of the training regimen’s intensity (see Figure 1). Similar to changes in fitness, neural specificity increased from pretest to posttest in some participants, and decreased in others, especially among those whose fitness did not improve. Overall there was no significant change in neural specificity with training (*F*_(1,45)_ = 1.891; *p* = 0.176; $\eta_{\text{p}}^{2}$ = 0.040) and no reliable interaction of neural specificity changes with group (*F*_(1,45)_ = 0.006; *p* = 0.936; $\eta_{\text{p}}^{2}$ = 0.000). Figure 2 displays mean changes in fitness and neural specificity.

To differentiate training-induced changes in neural specificity from changes in neural efficiency, we also tested whether training affected mean BOLD activation in stimulus-relevant regions, that is, the fusiform face area (FFA) for faces and parahippocampal place area (PPA) for buildings. We extracted the % BOLD signal change from four spherical ROIs (FFA left and right, PPA left and right, with 5 mm radius around center coordinates adapted from Ishai et al., 1999) for the appropriate condition (in FFA for faces, and in PPA for houses) using MarsBaR. We found a negative but non-significant relationship between change in fitness and change in BOLD activation in both the FFA (*r*_(46)_ = −0.201, *p* = 0.18, *R*^2^ = 0.040) and the PPA (*r*_(46)_ = −0.181, *p* = 0.23, *R*^2^ = 0.033). The average effect across both regions was also negative, but not significant (*r*_(46)_ = −0.234, *p* = 0.12, *R*^2^ = 0.055). For neither region alone, nor for the average, did we observe significant training-induced changes in % BOLD signal change (all *p* > 0.3), nor were there significant interactions with training group (all *p* > 0.2). All means and SD are provided in Table 2.

**Table 2:** **Pre- and post-training values (mean ± SD) for fitness measures, multivariate pattern analysis, and % BOLD signal change**.

	High intensity		Low intensity	
	Pre	Post	Pre	Post
VO_2_max (ml/min × kg)*^1^	21.26 (± 4.76)	23.32 (± 5.06)	21.95 (± 5.70)	22.34 (± 5.29)
VO_2_AT (ml/min × kg)*^2^	15.60 (± 3.18)	16.53 (± 3.49)	15.20 (± 4.25)	16.05 (± 3.57)
VO_2_*^3^	−0.13 (± 0.84)	0.19 (± 0.92)	−0.10 (± 1.07)	0.03 (± 0.96)
MVPA	0.11 (± 0.07)	0.09 (± 0.06)	0.10 (± 0.07)	0.09 (± 0.07)
% Bold signal change FFA	0.18 (± 0.14)	0.15 (± 0.17)	0.20 (± 0.14)	0.18 (± 0.18)
% Bold signal change PPA	0.25 (± 0.20)	0.20 (± 0.19)	0.19 (± 0.17)	0.20 (± 0.20)

Abbreviations: MVPA, Multivariate pattern analysis; FFA, fusiform face area; PPA, parahippocampal place area. *Indicates a significant main effect of time from the repeated measures ANOVA (p < 0.05). ^1^F_(1,49)_ = 4.805; p = 0.033; $\eta_{\text{p}}^{2}$ = 0.089. ^2^F_(1,50)_ = 4.902; p = 0.031; $\eta_{\text{p}}^{2}$ = 0.089. ^3^F_(1,49)_ = 5.637; p = 0.022; $\eta_{\text{p}}^{2}$ = 0.103.

## Discussion

In this study we investigated whether exercise-induced fitness improvements would be associated with enhanced neural specificity. We found a positive correlation between changes in fitness induced by a 6-month exercise intervention and changes in neural specificity, in the sense that participants whose physical fitness improved more also showed more positive changes in neural specificity. These data suggest that regular physical activity may reduce or even reverse aging-related declines in neural specificity that have been reported in earlier studies (Park et al., 2004, 2010; Payer et al., 2006).

In terms of mechanisms, we propose that regular exercise may counteract the age-related weakening of dopaminergic neuromodulation (Bäckman et al., 2006). Neurocomputational models predict that adequate DA availability helps to keep the sigmoidal gain function within ranges that optimize signal transmission (Li et al., 2001). Optimized signaling reduces the detrimental effects of neural noise, and leads to the generation of more distinct internal representations (Li and Sikström, 2002). We note that this hypothesis could be tested more directly using Positron Emission Tomography (PET) to investigate DA binding and neural specificity before and after an exercise intervention.

Age-related losses in dopaminergic neuromodulation have more often been reported in frontal regions than in VVC. However, there is evidence for age-related losses of DA in the human temporal, parietal, and occipital cortices as well (Kaasinen and Rinne, 2002), closely resembling the dopaminergic decline repeatedly observed in the striatum. Recently, Garrett et al. (2015) found effects of d-amphetamine (boosting DA levels) on BOLD signal variability in regions beyond typical DA projections (e.g., primary visual cortices). Moreover, in a PET study with 181 healthy adults between 64 and 68 years of age, Nyberg et al. (2016) found that both caudate and hippocampal D2 DA receptor availability were positively associated with episodic memory. These findings provide further evidence for the functional significance of DA across many regions of the human brain.

It is also possible that other neurotransmitters are playing a role, instead of, or in addition to, DA. For example, in a proton magnetic resonance spectroscopy study, Maddock et al. (2016) observed increased signals for both glutamate and GABA in the visual cortex following vigorous exercise, indicating increased glutamate and GABA levels. Likewise, animal work showed an increased visual response in mouse visual cortex during running using *in vivo* calcium imaging. The suggested mechanism includes disinhibition of glutamatergic pyramidal neurons through the interaction of two different GABAergic interneurons (Fu et al., 2014). However, these results were obtained during running and there is only preliminary evidence to suggest that more exercise in the preceding week also relates to higher resting glutamate (but not GABA) levels (Maddock et al., 2016). Furthermore, administration of GABA and a GABA agonist has been found to increase the orientation selectivity of individual visual neurons (increased neural specificity), while administration of a GABA antagonist has been found to decrease it (reduced neural specificity; Leventhal et al., 2003). Future work is needed to gauge the relative importance of different neurotransmitter systems, and their potential interactions, in mediating the effects of exercise on cognition.

Exercise has been shown to exert effects on other parameters of cerebral functioning, and these parameters may have contributed to our findings. Regarding fMRI, exercise-induced fitness changes were found to induce improved neural efficiency, that is, reductions (Colcombe et al., 2004; Voelcker-Rehage et al., 2011) or stability (Maffei et al., 2017) of BOLD activation in task-relevant areas as well as strengthened functional connections in relation to default mode and frontal executive networks (Voss et al., 2010). Though changes in neural efficiency, as investigated in these earlier studies, and changes in neural specificity, as investigated in the present study, can occur independently from each other, it seems worth exploring whether and in what way they are related empirically. Specifically, exercise training may lead to neural representations that achieve greater distinctiveness with a lower levels of neural activation.

Although our experimental task was not designed to look at neural efficiency, we were curious to see whether our training did affect mean BOLD activation in stimulus-relevant regions (FFA and PPA) and if so, in what way. Overall, we found negative, but non-significant, relationships between change in fitness and change in BOLD activation. These trends seem consistent with the hypothesis that improved fitness is associated with increased neural efficiency (i.e., less BOLD signal change). However, because we used a passive viewing task, there are no behavioral measures (e.g., reaction time, accuracy) from the scanning sessions that we could relate to the BOLD signal. We therefore acknowledge that the present study does not directly address neural efficiency, as this would require reduced BOLD activation in the absence of performance decrements.

At a more general level, exercise has been found to change brain perfusion (Pereira et al., 2007; Maass et al., 2015). Whereas Pereira et al. (2007) specifically looked at the hippocampus, Maass et al. (2015) also found fitness-related changes in perfusion to affect non-hippocampal cortical blood flow and blood volume. Changes in perfusion could potentially influence our results, as the fMRI BOLD signal measures a vascular response. Hence, we wondered whether the observed association might be due to changes in vascular response rather than changes in neural specificity. If so, one would expect to also observe an association between changes in mean activation level and changes in fitness. We therefore extracted mean activation values from VVC using MarsBaR, but found no reliable correlation with changes in fitness. Furthermore, vascular changes would not be expected to be limited to VVC, and so we tested whether associations between changes in fitness and changes in neural specificity were also present in brain regions that were not significantly activated by the task, such as medial orbitofrontal cortex, precentral gyrus, supramarginal gyrus, superior temporal gyrus, hippocampus, and parahippocampus. None of those regions, nor all of them combined, exhibited the change-change correlation observed in VVC. Each of these findings argues against a predominantly vascular interpretation of the observed effects. We note, however, that a calibrated fMRI approach would be needed to fully disentangle changes in neural activity from changes in vascular reactivity.

In contrast to Park et al. (2010), we did not observe associations between neural specificity and any of the cognitive tasks assessed in this study. One reason may be that Park et al. (2010) used a slightly different visual task in the fMRI scanner: Whereas our participants passively viewed single pictures, Park et al. (2010) presented two pictures side-by-side and asked their participants to make a same/different judgment, which may have introduced a demand characteristic that was absent in our task.

It is conceivable that exercise training as well as familiarization with the MR environment would alter participants’ intention to lie quietly in the scanner. To minimize differences in head movement between pretest and posttest, all participants took part in a Mock-Scanner session before pretest. In addition, we used the FMRIB Software Library (Jenkinson et al., 2012) to check for head motion artifacts using the aggregated measures dvars as well as framewise displacement (implemented in fsl_motion_outlier). We found no evidence for significant session differences in head motion.

Also, sample size as well as sample selectivity may have influenced our results. In a study with similar sample selection criteria, Voelcker-Rehage et al. (2015) noticed a comparably high amount of COMT met/met allele carriers. Since our participants were inactive but healthy and willing to change their lifestyle, it is rather likely that they were even more selective. Given the absence of genetic information in this study, this conjecture cannot be tested.

As discussed in Kleemeyer et al. (2016), the absence of group differences in fitness changes between the HI and the LI groups may indicate that the level of challenge provided by LI training was already effective in boosting fitness in sedentary older adults, thereby rendering the two training regimes similar to one another. The observed absence of group differences in fitness changes also provides a reasonable explanation for the absence of group differences in neural specificity changes. As for neural specificity, we did not observe a mean increase over time, but rather stability, as the numerical decrease was not statistically different from zero. We interpret this finding in terms of two opposing forces, one related to normal aging and the other related to the fitness intervention. On the one hand, neural specificity tends to decline with age. Participants did not escape normal aging while taking part in our study, and for this reason alone one would expect that neural specificity measured later in time is lower than neural specificity measured earlier in time. On the other hand, the training-induced fitness improvements apparently counteracted aging-related decrements in neural specificity, and apparently reduced or, in some cases, actually offset the effects of aging. We conclude that the effects of fitness improvements on brain functioning were not strong enough to result in a mean positive trend in neural specificity. Nevertheless, and in full agreement with our hypothesis, participants whose fitness improved more showed smaller declines, or even improvements, in neural specificity. From a design perspective, it would have been preferable to also include a no-contact control group in the study in order to document changes in fitness and neural specificity that would take place in the absence of any intervention.

To conclude, we found that exercise-related changes in fitness are positively associated with changes in neural specificity. Greater neural specificity is related to better fluid processing ability, so these results may explain some of the beneficial effects of exercise on cognition. Future longitudinal and intervention work should include higher intensity levels, longer training durations, and no-contact control groups to replicate and extend these findings.

## Author Contributions

MMK designed and conducted the study, analyzed the data, interpreted the results, and wrote the manuscript. TAP assisted with MVPA analysis, interpreted the results, and revised the manuscript. SS designed the study and revised the manuscript. NCB designed the neuroimaging protocol and revised the manuscript. LB performed the cardiovascular fitness assessment and revised the manuscript. UL designed the study, interpreted the results, and revised the manuscript.

## Funding

The work reported in this article was supported by the Max Planck Society and the German Research Foundation (DFG; Gottfried Wilhelm Leibniz Research Award 2010 to UL).

## Conflict of Interest Statement

The authors declare that the research was conducted in the absence of any commercial or financial relationships that could be construed as a potential conflict of interest.

## Footnotes

^1^http://www.fil.ion.ucl.ac.uk/spm/
